# Tofacitinib Ameliorates Retinal Vascular Leakage in a Murine Model of Diabetic Retinopathy with Type 2 Diabetes

**DOI:** 10.3390/ijms222111876

**Published:** 2021-11-02

**Authors:** Eimear M. Byrne, María Llorián-Salvador, Timothy J. Lyons, Mei Chen, Heping Xu

**Affiliations:** 1 Wellcome-Wolfson Institute for Experimental Medicine, School of Medicine, Dentistry and Biomedical Sciences, Queen’s University, Belfast 97 Lisburn Road, Belfast BT9 7BL, UK; ebyrne19@qub.ac.uk (E.M.B.); m.lloriansalvador@qub.ac.uk (M.L.-S.); lyonstj@musc.edu (T.J.L.); m.chen@qub.ac.uk (M.C.); 2 Department of Medicine, Division of Endocrinology, Diabetes & Metabolic Diseases, Medical University of South Carolina, Charleston, SC 29452, USA; 3 Diabetes Free South Carolina, Blue Cross Blue Shield of South Carolina, Columbia, SC 29229, USA

**Keywords:** JAK1, diabetes, JAK/STAT, retina, inflammation, macular edema, blood-retinal barrier

## Abstract

We have previously reported that inhibition of the Janus kinase 1 (JAK1) signaling ameliorates IL-17A-mediated blood-retinal barrier (BRB) dysfunction. Higher levels of IL-17A have been observed in the blood and intraocular fluids in patients with diabetic retinopathy (DR), in particular those with diabetic macular oedema. This study aimed to understand whether JAK1 inhibition could prevent BRB dysfunction in db/db mice, a model of type 2 diabetes (T2D). An in vitro study showed that high glucose treatment disrupted the junctional distribution of claudin-5 in bEnd3 cells and ZO-1 in ARPE19 cells and that tofacitinib citrate treatment prevented high glucose-mediated tight junction disruption. Albumin leakage, accompanied by increased levels of the phosphorylated form of JAK1 (pJAK1), was observed in three-month-old db/db mice. Treatment of two-and-a-half-month-old db/db mice with tofacitinib citrate for two weeks significantly reduced retinal albumin leakage and reduced pJAK1 expression. pJAK1 expression was also detected in human DR retina. Our results suggest that JAK1 inhibition can ameliorate BRB dysfunction in T2D, and JAK1 inhibitors such as tofacitinib citrate may be re-purposed for the management of diabetic macular oedema.

## 1. Introduction

Globally, 463 million people are affected by diabetes, and the number is predicted to rise to 700 million by 2045 [1]. Owing to its chronic nature, diabetes leads to many complications, including nephropathy, neuropathy, and retinopathy. Diabetic retinopathy (DR) is a complex complication that affects the retinal vasculature and neurons and can result in blindness. Diabetic macular oedema (DMO), in particular, is often associated with severe visual loss and occurs both in people with type 1 and 2 diabetes mellitus (T1DM, T2DM). As the global prevalence of T2DM is increasing rapidly, the number of people experiencing vision loss from DMO is rising [2,3]. Prevalence of DMO and DR increases with diabetes duration, and this is confounded by undiagnosed diabetes, which can lead to disease progression prior to clinical management of diabetes [4]. Blood retinal barrier (BRB) dysfunction and retinal microvascular degeneration are hallmarks of DR. The related retinal vascular leakage underpins the pathology of DMO. Current standards of care for DMO include the intraocular administration of anti-VEGF inhibitors, which have limited efficacy and require invasive repeat injections, or laser-photocoagulation, which can slow disease progression but cannot restore vision. Intravitreal injection of steroids or steroid implant (e.g., Ozurdex) have also been used to treat DMO, particularly for patients who do not respond to anti-VEGF therapy [5], although steroid-induced complications such as cataract and glaucoma limit the suitability of steroid-based treatments. More effective and safer therapies are urgently needed.

Inflammation has been implicated in the pathogenesis of diabetic complications in the retina, including DR and DMO. In diabetes, metabolic insults and dysregulated innate immune cell activation lead to a low-grade chronic inflammation, which drives BRB dysfunction [6]. The vitreous fluid levels of pro-inflammatory cytokines such as IL-6, MCP-1/CCL2, and ICAM-1 are related to DMO severity [7,8,9]. The JAK/STAT signalling pathway is a master regulator of cytokine signalling, and therefore, using an inhibitor of any of the JAK/STAT family members may not only be important in the context of direct inhibition of a JAK/STAT family member, but also in the regulation of downstream signals. In addition to previously reported roles for the JAK/STAT pathway in signalling from cytokines such as IL-6 [10] and VEGF [11], we recently reported IL-17A-JAK1-dependent BRB dysfunction [12]. Many cytokines/growth factors known to be involved in DR and DMO (e.g., VEGF, IL-6, IL-17A, etc.) are controlled by the JAK/STAT pathway [13,14]. For this reason, we hypothesised that targeting the JAK/STAT signaling pathway might be more effective than targeting one cytokine alone, such as with the use of anti-VEGF antibodies, particularly in the context of diabetes mediated BRB breakdown.

In this study, we investigated the effect of the JAK1 inhibitor tofacitinib citrate in DR-related BRB leakage in a mouse model of T2DM, the BKS.Cg-Dock7^m^+/+Lepr^db^J mice (referred as db/db in this paper). The db/db mice recapitulate several key hallmarks of diabetic retinopathy, including glial activation, neuroretinal thinning, ERG abnormalities and changes in visual function, GLAST downregulation [15,16], increased leukostasis and acellular capillaries [17], and vascular leakage [18]. We found that pJAK1 expression was increased in the retina of db/db mice, and that treatment with the JAK1/2 inhibitor tofacitinib citrate significantly reduced BRB leakage in these mice.

## 2. Results

### 2.1. Tofacitinib Citrate Protected iBRB and oBRB Tight Junctions under High-Glucose Conditions

**Figure 1:**
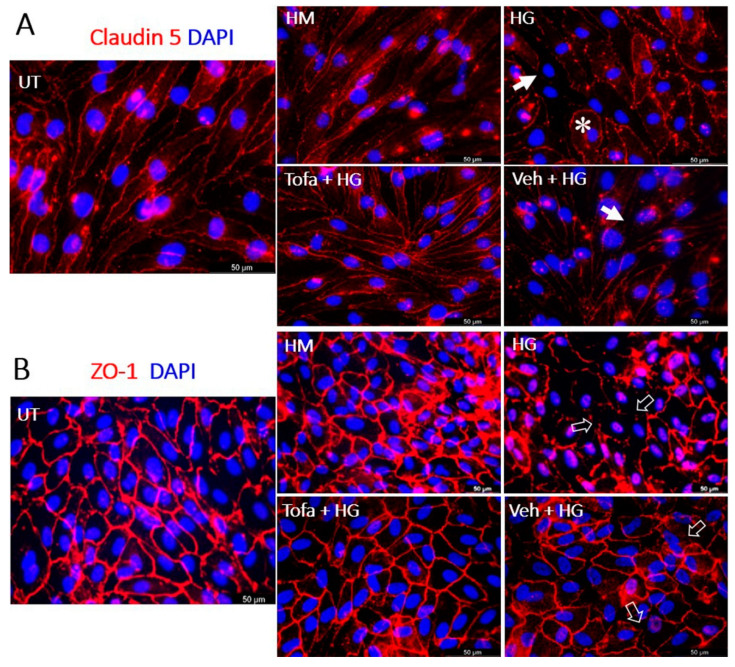
The effect of tofacitinib citrate on high glucose-induced tight junction dysmorphia. UT = untreated control. To mimic hyperglycaemia, cells were treated with physiologically high levels (25 mM) of D-glucose (HG) or D-mannitol (HM). Co-treatment with tofacitinib citrate (Tofa) (4.955 µM) or vehicle control (Veh) were used to investigate the effect of JAK1 inhibition on HG-induced junctional protein morphology. Immunostaining for cell nuclei (DAPI in blue) and junctional proteins (red) claudin 5 in bEnd.3 endothelial cells (**A**) and ZO-1 in ARPE-19 RPE cells (**B**). Arrows in (**A**) indicating disruption of claudin 5; asterisk in (**A**) indicating a rounded endothelium. Open arrows in (**B**) indicating ZO-1 disruption in ARPE19 cells. Representative images of *n = 3* independent experiments.

We used bEnd.3 endothelial and ARPE19 monolayer cultures as the inner BRB (iBRB) and outer BRB (oBRB) models to study the effect of tofacitinib citrate on high-glucose induced BRB damage. The iBRB structure was demonstrated using claudin-5 staining; whereas the ZO-1 was used to illustrate oBRB in ARPE19 cells (Figure 1). Under normal culture conditions, bEnd.3 cells have elongated morphology and claudin 5 junctions around the cell borders, whereas under 25 mM glucose (High Glucose, HG) treatment (three days), cell morphology became more rounded (asterisk, Figure 1A), and claudin 5 junction integrity was reduced (arrows, Figure 1A). Corresponding levels of D-mannitol (HM), used as an osmotic control, exhibited similar junctions to untreated cells. Cells treated with tofacitinib citrate and HG did not display the same damaged phenotype as cells treated with HG alone, while cells treated with vehicle and HG displayed a phenotype similar to HG-treated cells (Figure 1A).

Under normal culture conditions, ARPE-19 cells have cobblestone-morphology and ZO-1 junctions around the cell borders (Figure 1B), whereas under HG treatment (three days), this cobblestone morphology appeared disrupted (open arrow, Figure 1B). Co-treatment with tofacitinib citrate ameliorated this effect of HG on tight junctions. Vehicle control did not protect tight junctions from HG-mediated damage (open arrow, Figure 1B). The osmotic control (HM) had no effect on RPE tight junctions (Figure 1B).

### 2.2. Albumin Leakage in db/db Mice

**Figure 2:**
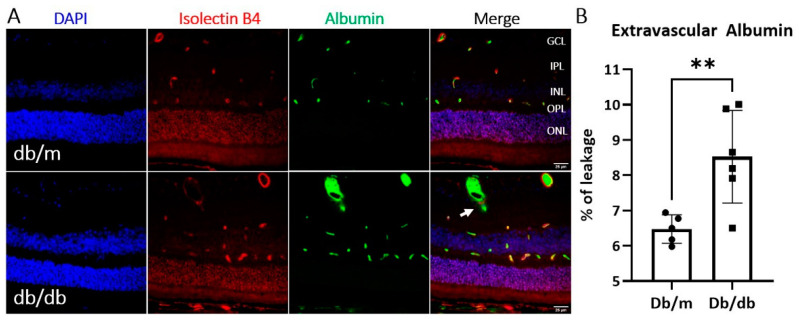
Albumin leakage in db/db and control db/m mice at 3 months of age. Immunostaining for leakage marker albumin and blood-vessel marker isolectin B4 were performed on retinal sections from 3-month old db/db and db/m mice. (**A**) Representative images of DAPI (blue), isolectin B4 (red) and albumin (green) staining in control (db/m) and diabetic (db/db) mice, *n* ≥ 5 animals per group. Arrow indicating extravascular albumin. GCL—ganglion cell layer; IPL—inner plexiform layer; INL—inner nuclear layer; OPL—outer plexiform layer; ONL—outer nuclear layer. (**B**) Quantification of albumin extravasation. Mean ± SD, ** *p* < 0.01, by unpaired *t*-test.

**Figure A1:**
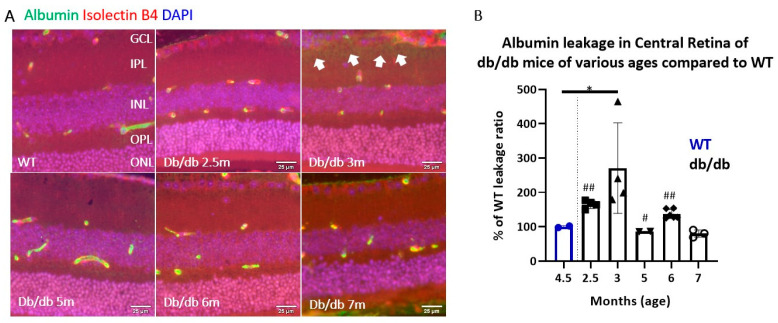
Albumin leakage in db/db mice of various ages. (**A**) Representative images of DAPI (blue), Isolectin B4 (red) and albumin (green) staining in 3-month old db/m (WT) and db/db mice of different ages (2.5, 3. 5, 6 and 7 months). (**B**) Quantification of albumin extravasation. Mean ± SD, *n* ≥ 2 mice per group. * *p* < 0.05 by One Way ANOVA. # *p* < 0.05, ## *p* < 0.01, compared to WT controls by unpaired *t*-test.

To understand at which time point BRB leakage occurred in the db/db mice, we conducted dual staining of vascular endothelial cells (isolectin B4) and albumin (an indicator of vascular leakage) in retinal sections collected from different ages of db/db (2.5–7 months) mice and wild-type mice. Albumin detected outside the endothelial cells was considered to indicate iBRB leakage (Figure A1A in Appendix A). Our results showed that db/db mice have elevated albumin leakage at three months of age (Figure A1B). Albumin leakage into the neuroretina at three-month-old db/db mice was confirmed to be significantly elevated compared to age-matched control non-diabetic heterozygous mice (Figure 2).

**Figure 3:**
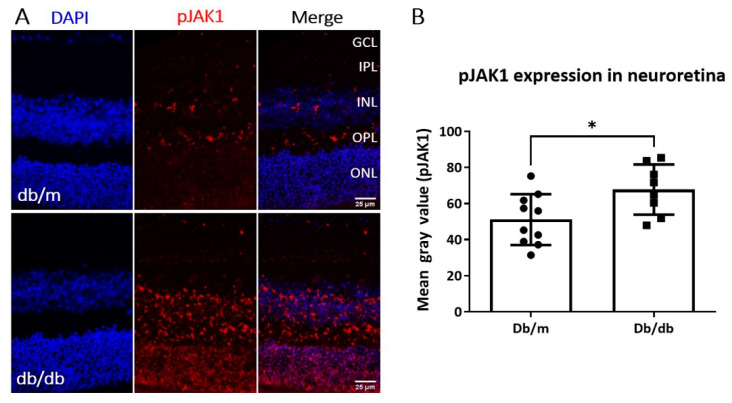
pJAK1 expression in 3-month old db/db mouse retinas. (**A**) Representative images of pJAK1(red) immunostaining, *n* ≥ 8 animals per group. GCL—ganglion cell layer; IPL—inner plexiform layer; INL—inner nuclear layer; OPL—outer plexiform layer; ONL—outer nuclear layer. (**B**) Quantification of pJAK1 expression in the neuroretina of db/db mice compared to heterozygous non-diabetic controls. Mean ± SD, * *p* < 0.05, by unpaired *t*-test.

Having shown BRB leakage in three-month-old db/db mice, we then examined pJAK1 expression in the neuroretinas of db/m and db/db mice (Figure 3). Indeed, elevated pJAK1 levels were found in the neuroretinas of db/db mice compared to db/m mice (Figure 3).

### 2.3. The Effect of Tofacitinib Citrate on Albumin Leakage in db/db Mice

**Figure 4:**
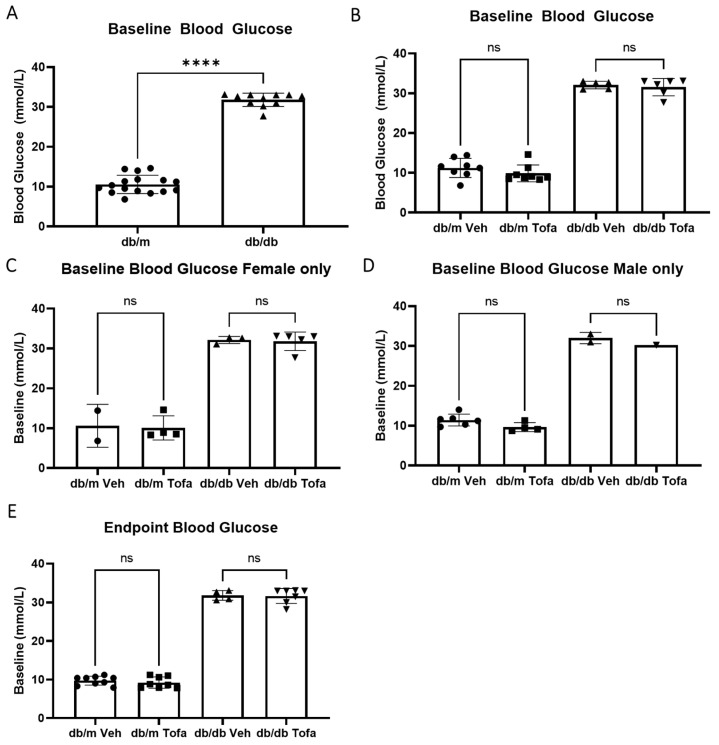
Tofacitinib citrate does not alter non-fasting blood glucose levels in db/db and db/m mice. Blood glucose measurements were taken from all mice between 2–3 pm at the beginning and end of the study. (**A**) db/db mice have higher levels of baseline blood-glucose than their db/m mice at 2.5 months of age. **** *p* < 0.0001 by Mann Whitney test. (**B**) Blood glucose levels in different groups of db/m and db/db mice before tofacitinib citrate (Tofa) or vehicle (Veh) treatment. (**C**,**D**) Blood glucose levels in female (**C**) and male (**D**) of different groups of db/m and db/db mice before tofacitinib citrate or vehicle treatment. (**E**) Endpoint blood glucose values in tofacitinib citrate or vehicle treated db/db and db/m mice. Mean ± SD, ns = not significant difference, Kruskall-Wallis test.

Having identified that pJAK1 levels were elevated in db/db mouse retinas, we then examined whether JAK1 inhibitor tofacitinib citrate could ameliorate BRB leakage in these mice. Firstly, we examined the effect of this inhibitor on blood glucose levels (Figure 4). The baseline glucose level was significantly higher in db/db mice than that in db/m mice (Figure 4A). There were no changes from baseline glucose following the two-week treatment with tofacitinib citrate, when sexes were analysed together (Figure 4B), or when female mice (Figure 4C) or male mice (Figure 4D) were analysed separately. As expected, the endpoint level of blood glucose in db/db mice was significantly higher than that db/m mice (Figure 4E).

**Figure 5:**
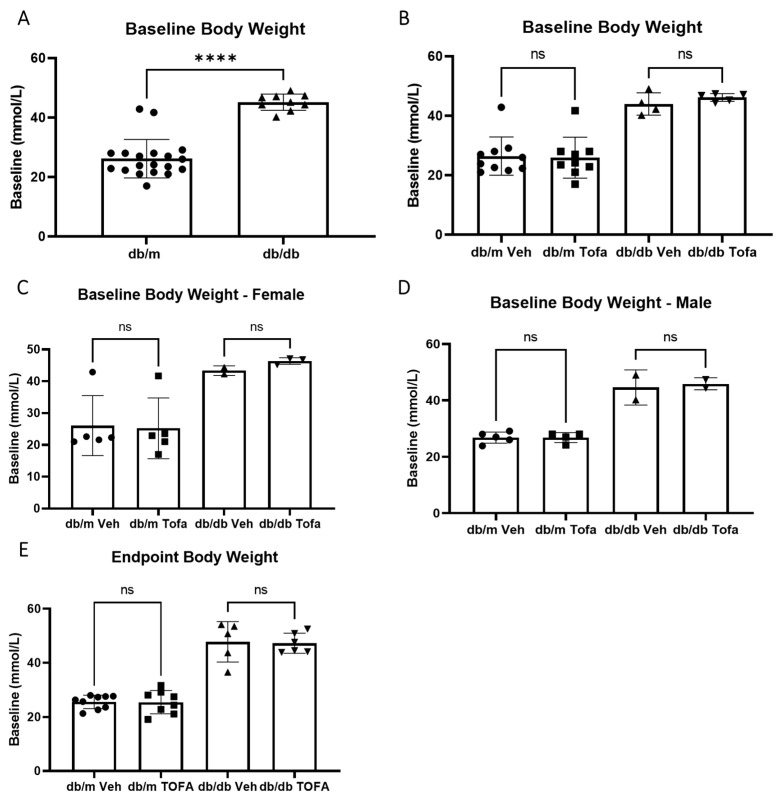
Tofacitinib citrate does not alter body weight in db/db or db/m mice. Blood weight measurements were taken from all mice daily throughout the study. (**A**) Baseline body weight in db/db and db/m mice at 2.5 months of age. **** *p* < 0.0001, Mann Whitney test. (**B**) Body weight in different groups of db/m and db/db mice before tofacitinib citrate or vehicle treatment. (**C**,**D**) Body weight in female (**C**) and male (**D**) of different groups of db/m and db/db mice before tofacitinib citrate (Tofa) or vehicle (Veh) treatment. (**E**) Endpoint body weight in tofacitinib citrate or vehicle treated db/db and db/m mice. Mean ± SD, ns = not significant difference, Kruskall-Wallis test.

In both male and female mice, there were no differences across the treatment or genotype groups in body weight, when measured as a percentage of the baseline for each animal. When gross body weight was compared, differences were only found between genotypes (diabetic obese versus non-diabetic non-obese, Figure 5A), but no differences were found between treatment groups (Tofacitinib citrate versus vehicle control, Figure 5B–D). Two-week treatment with tofacitinib or vehicle did not affect the body weight in db/db and db/m mice (Figure 5E). 

These data indicate that tofacitinib citrate is well tolerated by diabetic mice, and it does not seem to reduce BRB leakage via reduction of glycaemia.

**Figure 6:**
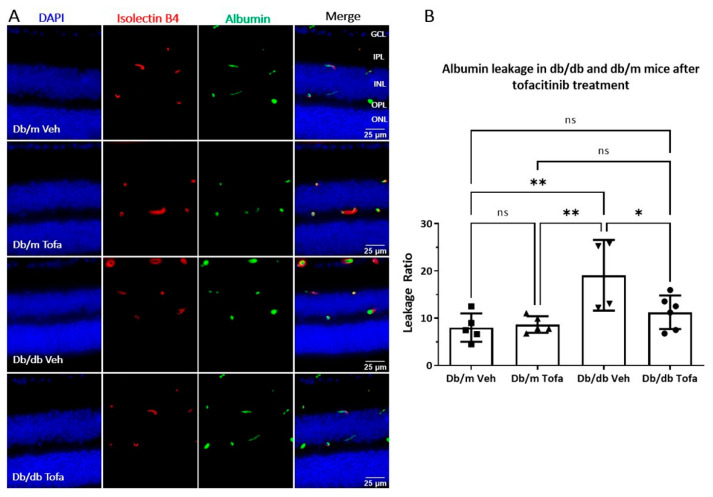
The effect of tofacitinib citrate on albumin leakage in db/db mice. Two and a half months old db/db and db/m mice were treated with tofacitinib citrate or vehicle for 2 weeks. Eyes were then collected and stained for isolectin B4 (red) and albumin (green). (**A**) Representative images showing isolectin B4 and albumin in db/m and db/db mice treated with either tofacitinib citrate or vehicle control. GCL—ganglion cell layer; IPL—inner plexiform layer; INL—inner nuclear layer; OPL—outer plexiform layer; ONL—outer nuclear layer. (**B**) Quantification of albumin extravasation. Mean ± SD, * *p* < 0.05, ** *p* < 0.01 by One Way ANOVA followed by Tukey′s multiple comparisons.

**Figure A2:**
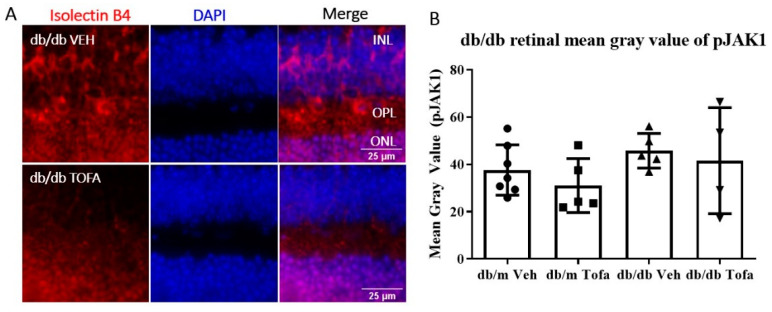
The effect of tofacitinib citrate treatment on pJAK1 expression in the retinas of db/db and db/m mice. 2.5-month old db/db and db/m mice were treated with tofacitinib daily for 2 weeks. Eyes were collected for pJAK1 staining. (**A**) Representative immunofluorescence of db/m and db/db retinas showing pJAK1 (red) and DAPI (blue). INL—inner nuclear layer; OPL—outer plexiform layer; ONL—outer nuclear layer. (**B**) Quantification of pJAK1 mean gray value in the neuroretina. Tofa = tofacitinib citrate, Veh = vehicle control.

Tofacitinib citrate treatment (daily for two weeks) reduced pJAK1 expression in the INL and OPL in the retina of three-month-old db/db mice (Figure A2). The treatment significantly reduced albumin leakage in db/db mice compared to vehicle control treatment, while it did not affect albumin leakage in non-diabetic mice (Figure 6).

### 2.4. pJAK1 Expression in the Human Diabetic Retina

**Figure 7:**
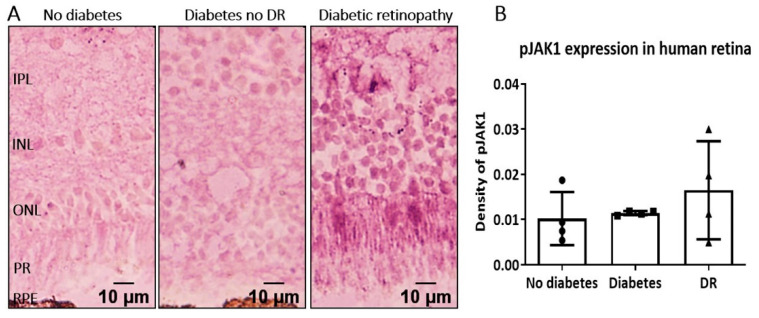
The expression of pJAK1 in human DR retinas. Retinal sections from non-diabetes, diabetes without retinopathy and diabetes with retinopathy were stained for pJAK1 (purple) and imaged by light microscopy. (**A**) Representative images from *n* = 4 patients per group, showing immunohistochemistry for pJAK1 in different groups of retinas. (**B**) Quantification of the density of pJAK1 in human retinas from different groups. Mean ± SD.

To understand the potential clinical relevance of pJAK1 as a target in DR management, we conducted a pilot study to examine pJAK1 expression in human retina samples from patients with no diabetes, diabetes but no clinical retinopathy, and diabetes complicated by retinopathy. pJAK1 was detected in one out of four retinas from non-diabetes donors and none in the retina from diabetes patients without DR. In two out of four DR patients, we saw increased pJAK1 staining in the neuroretina (Figure 7). These data indicate that at least a subset of DR patients have elevated pJAK1 in their neuroretinas and may therefore benefit from tofacitinib citrate treatment.

## 3. Discussion

In this study, we showed that the JAK1 inhibitor tofacitinib citrate preserved BRB integrity and reduced retinal vascular leakage in a model of T2DM. We also showed that pJAK1 expression was elevated in some DR patients compared to non-diabetic and people with diabetes without retinopathy. The JAK1/2 are normally activated by type 1 and type 2 cytokines [19]. We recently reported that a type 1 cytokine, IL-17A is capable of inducing pJAK1 expression in the retina [12]. Altered JAK1 signalling has been associated with T1DM previously, in studies that show JAK1 inhibition successfully ameliorated autoimmune diabetes in mice [20,21] and reduced insulin dependency in a patient with rheumatoid arthritis, systemic sclerosis, and T1DM [20]. In terms of diabetic complications, a JAK1 inhibitor baracitinib has been shown to protect the kidney from T2DM-induced albuminuria [22], highlighting a role for JAK1 in diabetes-induced vascular barrier dysfunction. Furthermore, gain-of-function in STAT3 mutations have been found to lead to T1DM [23]. Together, these studies highlight the potential of the JAK/STAT pathway as a therapeutic target in autoimmune diabetes and microvascular complications of both T1DM and T2DM.

Tofacitinib citrate selectively inhibits JAK1 and JAK3, and to a lesser extent than JAK2, and is approved by the European Medicines Agency and U.S. Food and Drug Administration for various autoimmune diseases, including rheumatoid arthritis, psoriatic arthritis, ulcerative colitis, and polyarticular juvenile idiopathic arthritis. Other JAK1 inhibitors, such as filgotinib, upadacitinib, peficitinib, and brepocitinib, have also been approved to treat autoimmune diseases [24,25,26]. The clinical approval of these drugs suggests that they are effective and safe and can be re-purposed for ocular use. A few studies have provided evidence of favorable ocular tolerance of tofacitinib citrate [27,28], as well as evidence of tofacitinib citrate in controlling ocular inflammation in dry eye disease [28,29,30], refractory uveitis and scleritis [31], and in spondyloarthritis-associated uveitis [32].

The JAK/STAT pathway is essential for the biological function of various cytokines and growth factors, the potential adverse effect of JAK1 inhibition on diabetes is an important consideration. One large clinical study compared the effects of tofacitinib citrate on diabetes worsening to other commonly used treatments for rheumatoid arthritis, namely abatacept, a TNF inhibitor (TNFi), rituximab, and tocilizumab. The authors found no difference in worsening of DM (measured as switching or intensification of treatment) with the use of the aforementioned four drugs, and a less of a risk in patients treated with tofacitinib citrate [33]. This was in line with our observation that tofacitinib treatment did not affect the blood glucose levels and body weight in T2D db/db mice.

Db/db mice are a widely used model of T2DM, and the mice age-dependently develop retinal neuronal and vascular degeneration–typical signs of DR [34,35]. A previous study reported no vascular leakage using fluorescence angiography in seven months old db/db mice [36]. In this study, we used sophisticated methods of detection, i.e., extravascular albumin leakage using fluorescence microscopy and Western blot, and observed significantly increased albumin leakage at three months of age, although the leakage declined after this time point. The increased retinal vascular leakage was accompanied by higher levels of pJAK1 expression, which may explain the therapeutic effect of tofacitinib citrate in our study. Importantly, we found that pJAK1 is also expressed in some human DR retinas. This observation suggests a likely role of pJAK1 in human DR. Future studies should aim to understand the link between pJAK1 expression and DMO in clinical settings and identify patients suitable for JAK1 targeted therapy.

In this study, we used intraperitoneal administration of tofacitinib citrate (15 mg/kg) in mice, which was well tolerated and was in line with previous studies [12,37,38]. Human patients currently receive tofacitinib citrate (Xelanjz) orally for the treatment of rheumatoid arthritis, ulcerative colitis amongst other inflammatory diseases. As diabetes is a systemic inflammatory disease, this administration route may be effective in DR, particularly for DMO management that requires immediate short-term intervention. However, for other DR-related pathologies, such as capillary degeneration, that require a lifetime management strategy, prolonged systemic inhibition of the JAK/STAT pathway may cause adverse effects, e.g., the risk of infection. For example, increased risk of fungal infection has been associated with long-term use of tofacitinib citrate [38]. Therefore, local (e.g., intravitreal) delivery of JAK1 inhibitors may be preferable.

## 4. Materials and Methods

### 4.1. Human Eye Tissue

Human eyes were obtained under a Material Transfer Agreement (MTA) with the University of Oklahoma (Oklahoma City, OK, USA). The study was approved by the Ethical Review Boards of Queen′s University Belfast (Approval date: 5 April 2017, Ref 17.08v2) and carried out within the parameters of the declaration of Helsinki. Human ocular tissues were stored in accordance with the Human Tissue Act (2004). Informed consent was waived because the human retinal sections were obtained, de-identified and postmortem, from NDRI (National Disease Research Interchange, Philadelphia, PA, USA), as previously described [39,40] and according to stipulated clinical criteria.

### 4.2. Cell Culture and Treatments

The human RPE cell line, ARPE-19 (ATCC^®^ CRL-2302™, Manassas, VA, USA) were cultured on glass coverslips in 24-well plates, in Dulbecco′s Modified Eagle Medium: Nutrient Mixture F-12 (DMEM/F12) (Cat. 11320033) supplemented with 10% FCS (Cat. 10270106) and 1% penicillin-streptomycin (Cat. 15140122) (all from Gibco, Waltham, MA, USA). For experiments, media was changed to lower serum (1% FCS) 24 h before treatment, to facilitate RPE cell quiescence. The mouse brain endothelial cell line, bEnd.3 cells (ATCC^®^ CRL-2299™) were cultured on glass coverslips in 24-well plates and maintained for experiments in DMEM with GlutaMAX™ Supplement (Gibco, Cat. 10566016) medium with 10% FCS and 1% penicillin-streptomycin, of same origins as above. An additional 25 μM D-glucose (Cat. G8769, Sigma-Aldrich, St. Louis, MO, USA) or osmotic control D-mannitol (Cat. M4125, Sigma-Aldrich) were added to culture medium for experiments. Tofacitinib citrate (Sigma, Cat. PZ0017) (25 mg) was dissolved in 100 µL dimethylsulfoxide (DMSO, Cat. No. D8418 Sigma-Aldrich, St. Louis, MO, USA) and further diluted in PBS immediately before use to 2.5 µg/mL (4.955 µM). Vehicle control for tofacitinib was DMSO diluted 1:100,000 in PBS, i.e., 0.00001% DMSO. Cells were pre-treated with tofacitinib citrate for 30 min before the addition of high glucose and tofacitinib citrate as a co-treatment for 3 days (endothelial cells) and 6 days (RPE cells).

### 4.3. Immunocytochemistry

Following high glucose or control treatment, with or without tofacitinib citrate, ARPE-19 and bEnd.3 cells were stained for rabbit anti-ZO-1, rabbit anti-Claudin 5 respectively, to examine tight junction alterations. Cells were fixed in 2% PFA or ice-cold methanol respectively for 10 min. For methanol-fixed cells, no permeabilisation was required, and for PFA-fixed cells, 0.1% Triton X was used in the blocking buffer, which was 5% Donkey Serum for all experiments. Antibodies were incubated overnight at 4 degrees. The next day, coverslips were thoroughly washed prior to incubation with secondary antibody diluted 1:300 in blocking buffer for 1 h at room temperature. Following washing, coverslips were mounted using Vectashield containing DAPI and imaged using Leica Dmi8 microscope.

### 4.4. Animal Care and Housing

The BKS.Cg-Dock7^m^+/+Lepr^db^J and the heterozygotes from the colony BKS.Cg-Dock7^m^+/+Lepr^db/+^ (referred as db/db and db/m respectively in this paper) mice (Jackson Laboratory, Bar Harbor, ME, USA), aged 2.5, 3, 5 and 6 months old, of both sexes were used for these studies, *n* ≥ 6 animals of each genotype were assigned per experimental group (tofacitinib citrate versus vehicle). Mice were maintained in the Biological Services Unit at Queen′s University Belfast with free access to food and water on a 12 h light/dark cycle, in accordance with the ARVO Statement for the Use of Animals in Ophthalmic and Vision Research. All procedures were approved by the UK Home Office Animals (Scientific Procedures) Act 1986, and the local animal welfare ethical review board of Queen′s University Belfast. Mice were monitored routinely for changes in blood glucose and body weight. Blood glucose measurements were taken from tail blood using the SD Code free Blood Glucose Meter (SD Biosensor Inc., Irvine, CA, USA).

### 4.5. Tofacitinib Citrate Administration

2.5-month-old db/db and db/m mice (*n* > 6 per group) were treated with tofacitinib citrate (15 mg/kg, i.p. in 100 µL) once daily for two weeks. This dose and route of tofacitinib citrate administration was based on previous studies from us and others and was proven to be well-tolerated and effective [12,37,38]. The age-matched db/db mice in control group received the same volume of vehicle (0.01% DMSO) daily for 2 weeks.

Blood glucose levels and body weight were measured before and after treatment. At endpoint, eyes were collected and processed for analyses of albumin leakage and pJAK1 expression (see below).

### 4.6. Processing of Mouse Eyes

Paraffin-embedded eyes were sectioned at 5 µm thickness. De-waxing was carried out by immersing slides in 3 changes of clearene for 5 min each, followed by 3 changes of 100% ethanol for 3 min each, and followed by 5 min in running water. Citraconic anhydride (Sigma, Cat. 125318) pH 7.4 at 95 °C for 30 min was used for antigen retrieval.

### 4.7. Albumin Staining & Quantification

Mouse eye sections were incubated with goat anti-albumin and biotinylated Griffonia Simplicifolia Lectin I Isolectin B4 (Table 1) overnight at 4 °C. The next day, slides were washed in PBS prior to incubation with appropriate secondary antibodies. Slides were mounted with DAPI-Vectashield and imaged using Leica DMi8 epifluorescence microscope. Images were analysed using FIJI (National Institutes of Health, Bethesda, MD, USA), Isolectin B4-positive ROIs were restored on the albumin channel and measured, prior to whole neuroretina measurements. Leakage ratio was calculated as follows:

Leakage Ratio = (Extravascular albumin)/(Total albumin in neuroretina)

### 4.8. pJAK1 Staining & Quantification

Mouse eye sections were blocked with 5% donkey serum for 1 h at room temperature, followed by incubation with anti-pJAK1 antibody (1:100) overnight at 4 °C. After several washes, samples were incubated with donkey anti-rabbit 594 (1:300, Stratech Scientific Ltd., Ely, UK) for 2 h. Slides were washed, mounted with DAPI-vectashield, and imaged as above. pJAK1 in the neuroretina was quantified using FIJI.

For human eyes, paraffin-embedded eye sections were melted in a histology oven, before de-waxing. Antigen retrieval with citraconic anhydride was carried out as described above. Eye sections were permeabilised for 15 min with 2% Triton X, and blocked for 12 min with BLOXALL (Vector Labs). The samples were incubated with pJAK1 antibody (1:50, Invitrogen) overnight at 4 °C. Slides were washed 3 times in PBS followed by incubation with anti-rabbit HRP (1:100, Abcam) for 2 h at room temperature. After thorough washing, slides were incubated with VIP solution (1 drop of reagents 1, 2, 3 and 4 in 1.66 mL PBS) (Vector Labs) for 6 min at room temperature. Slides were put through 3 × 5 min ethanol and 3 × 5 min clearene before mounting in DPX (Sigma). Samples were imaged using Leica Dmi8 microscope.

### 4.9. Statistical Analyses

Graph generation and statistical analyses were performed using GraphPad Prism 9 (GraphPad Software Inc., San Diego, CA, USA). The Mann-Whitney test was used to compare difference between two groups and three or more groups were analysed using One-way ANOVA followed by Tukey′s multiple comparisons.

## 5. Conclusions

JAK1 activation is involved in BRB dysfunction in db/db mice and inhibition of pJAK1 with tofacitinib citrate ameliorated retinal vascular leakage in db/db mice. Targeting pJAK1 may be a novel approach to treat diabetic macular oedema.

## Figures and Tables

**Table 1:** Antibodies used for immunostaining.

Target	Company, Product Number	Dilution Used
ZO-1	Thermofisher, 61-7300	1:50 (IF)
Claudin 5	Thermofisher, 34-1600	1:50 (IF)
Phospho-JAK1 (Tyr1034, Tyr1035)	Thermofisher, PA5-104554	1:50 (IF, IHC-P)
Albumin	Bethyl, a90-134a	1:800 (IHC-p), 1:1000 (WB)
Biotinylated Isolectin B4	Vector Labs, VEC.B-1205	1:50 (IHC-P)
Alexa Fluor^®^ 594 AffiniPure donkey anti-rabbit IgG (H + L)	Stratech, 711-585-152	1:300 (IF), 1:300 (IHC-p)
Donkey anti-rabbit 488	Thermofisher, 34-1600	1:50 (IF)
Streptavidin, Alexa Fluor™ 594 conjugate	Thermofisher, S11227	1:300 (IHC-p)
Alexa Fluor^®^ 488 AffiniPure donkey anti-goat IgG (H + L)	Stratech, 705-545-147	1:300 (IHC-p)

IF: immunofluorescence; IHC: Immunohistochemistry-paraffin.

## Data Availability

The data presented in this study are all contained within the main body of this article.
